# Time-varying cost-effectiveness analysis of sodium-glucose cotransporter-2 inhibitors in Chinese patients with heart failure and reduced ejection fraction: A microsimulation of the real-world population

**DOI:** 10.3389/fphar.2025.1527972

**Published:** 2025-02-25

**Authors:** Xinyu Zou, Xingchen He, Qingyang Shi, Si Wang, Nan Li, Yiling Zhou, Ming Hu, Li Luo, Yiwen Shen, Ye Zhu, Chim C. Lang, Zhiming Zhu, Haoming Tian, Sheyu Li

**Affiliations:** ^1^ Department of Endocrinology and Metabolism, MAGIC China Center, West China Hospital of Sichuan University, Chengdu, China; ^2^ Faculty of Science and Engineering, University of Groningen, Groningen, Netherlands; ^3^ Department of Cardiology, West China Hospital of Sichuan University, Chengdu, China; ^4^ Department of Informatics, West China Hospital of Sichuan University, Chengdu, China; ^5^ West China School of Pharmacy, Sichuan University, Chengdu, China; ^6^ Business School, Sichuan University, Chengdu, China; ^7^ School of Business and Management, The Hong Kong University of Science and Technology, Hong Kong, Hong Kong SAR, China; ^8^ Division of Molecular and Clinical Medicine, Ninewells Hospital, University of Dundee, Dundee, United Kingdom; ^9^ Department of Hypertension and Endocrinology, Center for Hypertension and Metabolic Diseases, Daping Hospital, Army Medical University, Chongqing, China

**Keywords:** cost-effectiveness, heart failure, microsimulation, sodium-glucose cotransporter-2 inhibitors, time-varying

## Abstract

**Objective:**

Sodium-glucose cotransporter-2 (SGLT2) inhibitors showed time-varying effects in heart failure and reduced ejection fraction (HFrEF), but corresponding cost-effectiveness in different timeframes remained poorly understood. This study estimated the time-varying cost-effectiveness of SGLT2 inhibitors in HFrEF from the perspective of the Chinese healthcare system.

**Methods:**

Based on real-world individual patient data, a 2-year microsimulation model was constructed to evaluate the cost-effectiveness of adding SGLT2 inhibitors to standard therapy compared with standard therapy alone among patients with HFrEF. A published prediction model informed transition probabilities for all-cause death and hospitalization for heart failure. The time-varying effects of SGLT2 inhibitors, medical costs, and utility values were derived from the published literature. Scenario analyses in different timeframes were conducted to assess the trend of cost-effectiveness over time.

**Results:**

Compared with standard therapy alone, SGLT2 inhibitors plus standard therapy were found cost-effective at a willingness‐to‐pay (WTP) threshold of $12,741 per quality‐adjusted life year (QALY) gained in 2 years. The incremental cost-effectiveness ratio (ICER) decreased from $12,346.07/QALY at 0.5 years to $9,355.66/QALY at 2 years. One-direction sensitivity analysis demonstrated that the cost-effectiveness of SGLT2 inhibitors was most sensitive to the cost of SGLT2 inhibitors, the cost of hospitalization for heart failure, the cost of standard therapy for heart failure, and the baseline risks of all-cause death and hospitalization for heart failure. Probabilistic sensitivity analysis proved the robustness of the results.

**Conclusion:**

Adding SGLT2 inhibitors to standard therapy was found to be cost-effective in Chinese patients with HFrEF. Longer treatment appeared to be more economically favorable, but further explorations are warranted.

## 1 Introduction

Heart failure is a heterogeneous, progressive clinical syndrome that affects 64 million people worldwide and costs $108 billion annually (GBD, 2017 Disease and Injury Incidence and Prevalence Collaborators, 2018; Cook et al., 2014). The 5-year survival rate for chronic heart failure ranged from 51.5% to 63% (Jones et al., 2017). Approximately 13.7 million adults aged ≥35 years experience heart failure in China, and the total medical cost for heart failure in 2012 was $5.4 billion (Cook et al., 2014; Hao et al., 2019). Despite several advances in heart failure treatment in recent years, heart failure remains a major health concern in China because of the increasing prevalence and suboptimal management (National Center for Medical Care Quality Control of Cardiovascular Diseases, 2023). People with heart failure experience considerable residual risks for cardiovascular events and resulting death, with recurrent hospitalizations for worsening heart failure being the leading contributor to impaired quality of life and economic burden. Furthermore, 31.9% of patients hospitalized due to heart failure and reduced ejection fraction (HFrEF) died within 3 years after discharge (Wang et al., 2024).

Sodium-glucose cotransporter-2 (SGLT2) inhibitors proved efficacious for people with heart failure regardless of the presence of type 2 diabetes (Zou et al., 2024; Zou et al., 2022). According to the recommendations of the 2023 Focused Update of the 2021 European Society of Cardiology Guidelines for the diagnosis and treatment of acute and chronic heart failure, patients with chronic heart failure should receive SGLT2 inhibitors to reduce hospitalization for heart failure or cardiovascular death (McDonagh et al., 2023).

Previous experience revealed an unsatisfactory delay before patients with heart failure achieved guideline-directed medical therapy (GDMT) (Greene et al., 2018). In the US, only one in five eligible patients with HFrEF received SGLT2 inhibitors at discharge (Pierce et al., 2023). The usage rate of SGLT2 inhibitors in Chinese patients with HFrEF has gradually increased to 64.4% in 2023 but remains suboptimal according to the recommended guidelines (Zhang et al., 2024). As SGLT2 inhibitors receive the top recommendation for heart failure, understanding the region-specific cost-effectiveness of the agent is crucial in translating research findings into clinical practice. Previous cost-effectiveness analyses based on cohort-based Markov models demonstrated that adding SGLT2 inhibitors to standard therapy was cost-effective in people with HFrEF from the perspective of the Chinese healthcare system (Yao et al., 2020; Jiang et al., 2022; Lin et al., 2022; Sang et al., 2022; Tang and Sang, 2022). The adjunct use of SGLT2 inhibitors to standard therapy for HFrEF yielded an incremental cost-effectiveness ratio (ICER) ranging from $1,893.59 to $6,946.69 per quality-adjusted life year (QALY) gained. However, Markov models could not capture the heterogeneity across different individuals and the legacy of events after baseline (Rutter et al., 2011). Since the relative effects of SGLT2 inhibitors in preventing hospitalization due to heart failure vary over time, the cost-effectiveness estimates from Markov models may hardly show the reality (Zou et al., 2024). Notably, the cost-effectiveness of SGLT2 inhibitors in patients with heart failure needs to be reassessed with up-to-date evidence.

Using a real-world population with HFrEF and considering individual characteristics, this microsimulation study reassessed the time-specific cost-effectiveness of adding SGLT2 inhibitors to standard therapy in Chinese patients with HFrEF.

## 2 Materials and methods

From the perspective of the Chinese healthcare system, the conduction and reporting of this study followed the Consolidated Health Economic Evaluation Reporting Standards 2022 (CHEERS 2022) statement (Husereau et al., 2022).

### 2.1 Model population

The inpatient electronic medical records (EMRs) of a general tertiary care hospital in southwestern China were screened, and the eligible patients with HFrEF discharged between 1 January 2011 and 30 September 2018 were retrospectively recruited. HFrEF was identified by documented echocardiographic evidence of a left ventricular ejection fraction (LVEF) ≤40%. Complete data of major prognostic factors, diagnoses at discharge, and prescriptions were required to define the initial health state and predict clinical prognosis (as detailed in Supplementary Appendix SA1). Among 1,256,014 inpatients, 4,227 met the eligibility criteria (Table 1; Supplementary Appendix SA2). Our previous study described the characteristics of the study population (Zhou et al., 2021).

**TABLE 1:** Continuous variables below were described with median (IQR).

	Overall (n = 4,277)
Age, median (IQR), years	63 (52, 72)
Female, No. (%)	1,132 (26.8)
Heart rate, median (IQR), beats/minute^a^	76 (70, 84)
SBP, median (IQR), mm Hg	120 (108, 131)
DBP, median (IQR), mm Hg^a^	68 (60, 77)
Alcohol use, No. (%)	1,652 (39.1)
Smoking, No. (%)	
No	2,205 (52.2)
Current	764 (18.0)
Past	1,258 (29.8)
Peripheral edema, No. (%)	613 (14.5)
LVEF, median (IQR), %	33 (28, 37)
Distribution, No. (%)	
30%–40%	2,864 (67.8)
20%–29%	1,219 (28.8)
≤19%	144 (3.4)
NYHA classification, No. (%)	
I	690 (16.3)
II	740 (17.5)
III	1,792 (42.4)
IV	1,005 (23.8)
eGFR, median (IQR), mL/min/1.73 m^2^	79.90 (62.64, 95.50)
BUN, median (IQR), mmol/L	7.00 (5.40, 9.00)
Hemoglobin, median (IQR), g/L	132 (116, 146)
NT-proBNP, median (IQR), pg/mL	16,449 (1,976, 35,000)
HbA1c, median (IQR), %^a^	5.29 (4.72, 6.27)
Medical history, No. (%)	
Diabetes mellitus	942 (22.3)
Chronic obstructive pulmonary disease	723 (17.1)
Hypertension	1,541 (36.5)
Acute myocardial infarction	625 (14.8)
Ischemic heart disease	1,954 (46.2)
Rheumatic heart disease	391 (9.3)
Cardiomyopathy	1,224 (29.0)
Arrhythmia	1,677 (39.7)
Medical treatment, No. (%)	
Diuretics^b^	2,911 (68.9)
Beta-blockers	2,965 (70.1)
RAAS inhibitors	1,596 (37.8)
Statins	779 (18.4)

^a^ Heart rate, diastolic blood pressure, and glycated hemoglobin were available in 4,035, 4,217, and 4,178 patients, respectively.

^b^ Diuretics included loop diuretics and spironolactone.

Abbreviations: BUN: blood urea nitrogen; DBP: diastolic blood pressure; eGFR: estimated glomerular rate filtration; HbA1c: glycated hemoglobin; LVEF: left ventricular ejection fraction; NT-proBNP: N-terminal pro-B-type natriuretic peptide; NYHA: New York heart association; RAAS: renin–angiotensin-aldosterone system; SBP: systolic blood pressure.

We collected patient-level demographic information at admission, final values of vital signs, laboratory test results, left ventricular ejection fraction (LVEF), and prescription records during the hospital stay as baseline characteristics of the model population. Obtention of prior patient consent was waived because data collection was based on the EMR system retrospectively.

### 2.2 Model overview

**FIGURE 1:**
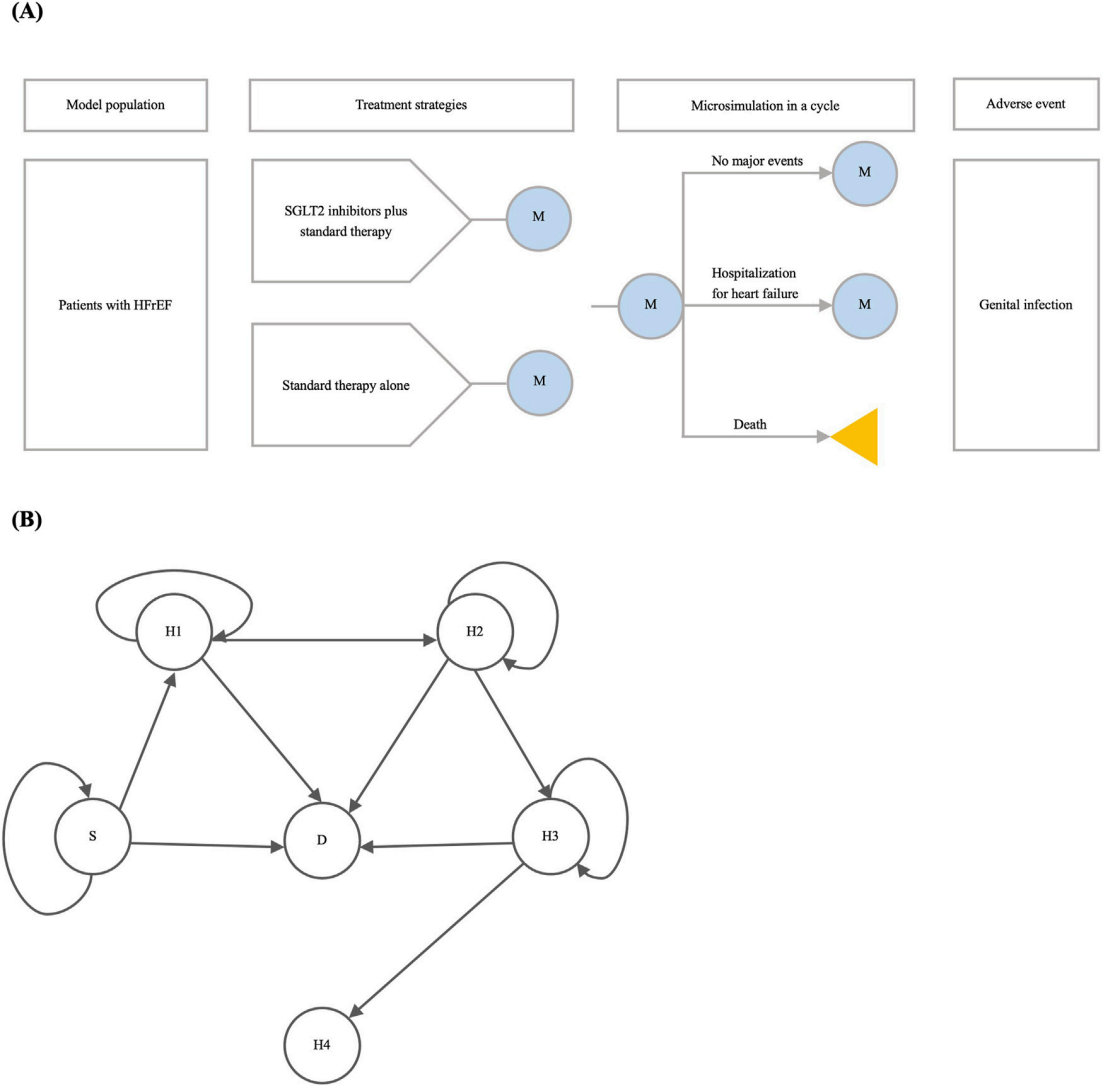
The microsimulation model. Notes: **(A)** shows the overview of the model. M indicates the microsimulation process within a cycle. The yellow triangle indicates that the simulation terminates once the patient dies; **(B)** shows the possible state transition of the model in 2 years. Abbreviations: D, death; H1: first hospitalization for heart failure; H2: second hospitalization for heart failure; H3: third hospitalization for heart failure; H4: fourth hospitalization for heart failure; HFrEF: heart failure and reduced ejection fraction; S: stable heart failure state; SGLT2: sodium-glucose cotransporter-2.

We constructed a state-transition microsimulation model to estimate the time-varying clinical impact and cost-effectiveness of adding SGLT2 inhibitors to standard therapy compared with standard therapy alone in people with HFrEF (Figure 1). The time horizon of the model was 2 years, with 6 months as a cycle. The longer time horizon was beyond the scope of the proposed model because of the lack of long-term evidence. A well-established and validated risk predictor facilitated some individual-level risk estimates for all-cause death and hospitalization for heart failure (Voors et al., 2017). The efficacy of SGLT2 inhibitors, utility values, and medical expenditures were derived from meta-analysis, cohort studies, and publicly available Chinese sources. We assumed input parameters extracted from the non-Chinese population were consistent with those of the Chinese population and evaluated their impact in the sensitivity analyses. The establishment of the model drew inspiration from the microsimulation tutorial by the Decision Analysis in R for Technologies in Health (DARTH) workgroup, and it was built in R version 4.0.2 (Krijkamp et al., 2018).

### 2.3 Treatment strategies

The clinical courses of the model population under two treatment strategies were simulated (Figure 1). The two strategies are as follows: 1) adding SGLT2 inhibitors approved for heart failure in China (i.e., dapagliflozin and empagliflozin) to standard therapy; 2) standard therapy alone. Standard therapy here referred to usual care for HFrEF without SGLT2 inhibitors, which consisted of GDMTs for HFrEF (or, angiotensin-converting enzyme inhibitors, angiotensin receptor blockers, mineralocorticoid receptor antagonists, and beta blockers), other drugs, surgical procedures, and diagnostics (Huang et al., 2017).

### 2.4 Clinical events

Clinical events in the proposed model included major events (all-cause death and hospitalization for heart failure) and adverse events (genital infection) (Figure 1). Six independent health states within four cycles were established based on the occurrence of all-cause death and the number of hospitalizations for heart failure: 1) stable heart failure; 2) first hospitalization for heart failure; 3) second hospitalization for heart failure; 4) third hospitalization for heart failure; 5) fourth hospitalization for heart failure; 6) death. The initial state of the model population was a stable heart failure state. The transition probabilities between health states within each cycle were determined by predicted prognostic risk under standard therapy alone and the efficacy of SGLT2 inhibitors. To mitigate the population diversity during the implementation of the risk calculator in a different population, the prediction model was calibrated using a Chinese cohort of heart failure (Huang et al., 2021). Because hospitalization for heart failure in the past year was a predictor of the risk of hospitalization for heart failure but not all-cause death (Voors et al., 2017), we assumed the risk of hospitalization for heart failure in later cycles varied according to the number of hospitalizations for heart failure in preceding cycles, and the risk of all-cause death was not impacted by previous hospitalizations. This microsimulation considered only genital infection due to scarcity of other safety issues. We extracted the incidence rates of genital infection from a UK cohort study and converted these rates to cycle-length transition probabilities (Hirji et al., 2012). The transition probabilities of genital infection varied depending on sex and diabetes status and involved the legacy of previous genital infections on future ones. Once the patient died, the simulation terminated subsequently. More details are provided in Supplementary Appendix SA1 in the Supplementary Material.

### 2.5 Efficacy of SGLT2 inhibitors

The health effects of SGLT2 inhibitors were modeled by applying the time-varying relative effects of SGLT2 inhibitors from a meta-analysis to the risks under standard therapy alone (Zou et al., 2024). As the benefit of SGLT2 inhibitors on death may be diluted by non-cardiovascular death, the risk ratios of cardiovascular death were determined to estimate the impact on all-cause death. The time-varying risk ratios of all-cause death were 0.87 (95% confidence interval [CI], 0.78–0.96) in the first cycle, 0.88 (95% CI, 0.78–0.98) in the second cycle, 0.89 (95% CI, 0.82–0.96) in the third cycle, and 0.89 (95% CI, 0.82–0.96) in the fourth cycle. SGLT2 inhibitors reduced hospitalization for heart failure by 39% (95% CI, 44%–34%) in the first cycle, 33% (95% CI, 35%–30%) in the second cycle, 28% (95% CI, 31%–26%) in the third cycle, and 25% (95% CI, 29%–21%) in the fourth cycle. The relative effect of SGLT2 inhibitors on genital infection remained unchanged over time (risk ratio 2.23, 95% CI 1.53 to 3.24, Table 2).

**TABLE 2:** Model input parameters.

	Mean value	Standard error	Distribution
Transition probabilities per cycle			
Regression coefficients of predictive variables for all-cause death^a^, (Voors et al., 2017)			Normal
Age, years	0.020	0.005	
Hemoglobin, g/dL	−0.117	0.023	
Log-BUN, mmol/L	0.419	0.062	
Log-NT-proBNP, ng/L	0.336	0.044	
Beta-blocker use at baseline	−0.274	0.087	
Regression coefficients of predictive variables for hospitalization for heart failure^a^ ^,^ (Voors et al., 2017)			Normal
Age, years	0.010	0.005	
SBP, mm Hg	−0.010	0.000	
Peripheral edema	0.432	0.082	
eGFR, mL/min/1.73 m^2^	0.548	0.084	
Hospitalization for heart failure in last year	−0.010	0.000	
Probabilities per cycle for genital infection^b^ ^,^ (Hirji et al., 2012)			Log-normal
Men without diabetes and history of genital infection	0.001	0.086	
Women without diabetes and history of genital infection	0.005	0.040	
Men with diabetes without history of genital infection	0.004	NA	
Women with diabetes without history of genital infection	0.009	NA	
Men with history of genital infection without diabetes	0.078	NA	
Women with history of genital infection without diabetes	0.091	NA	
Men with diabetes and history of genital infection	0.040	NA	
Women with diabetes and history of genital infection	0.065	NA	
Effect of SGLT2 inhibitors, risk ratio^c^(Zou et al., 2024)			Log-normal
All-cause death			
First cycle	0.87	0.053	
Second cycle	0.88	0.058	
Third cycle	0.89	0.040	
Fourth cycle	0.89	0.040	
Hospitalization for heart failure			
First cycle	0.61	0.042	
Second cycle	0.67	0.019	
Third cycle	0.72	0.018	
Fourth cycle	0.75	0.027	
Genital infection	2.23	0.191	
Utilities of health states			Normal
Heart failure (Göhler et al., 2009)			
NYHA class Ⅰ	0.855	0.005	
NYHA class Ⅱ	0.771	0.005	
NYHA class Ⅲ	0.673	0.006	
NYHA class Ⅳ	0.532	0.027	
Disutilities per event			Normal
Hospitalization for heart failure (Göhler et al., 2009)			
1 rehospitalization	−0.024	0.007	
2 rehospitalizations	−0.031	0.009	
≥3 rehospitalizations	−0.055	0.001	
Genital infection (Barry et al., 1997)	−0.003	0.001	
Costs ($)^d^			NA
Cost of standard HF therapy per cycle (Huang et al., 2017)	2,656.21	NA	
Cost of SGLT2 inhibitors per cycle (Sichuan Pharmaceutical Equipment Bidding and Purchasing Service Center, 2024)	116.67	NA	
Cost of hospitalization for HF per event (National Health Commission of the People’s Republic of China, 2022)	13,94.86	NA	
Cost of genital infection per event^e^	49.31	NA	
Discount rate for QALYs and costs	3%	NA	NA

^a^ Transition probabilities for all-cause death and hospitalization for heart failure for patients under standard therapy alone in the first cycle were predicted by the BIOSTAT-CHF prediction model based on individual baseline characteristics. The mean values of regression coefficients and corresponding standard errors for predictive variables were converted from reported hazard ratios and 95% confidence intervals. More details are provided in Supplementary Appendix SA1.

^b^ We derived the transition probabilities for genital infection per cycle from a UK cohort study. More details are provided in Supplementary Appendix SA1.

^c^ The relative effects of SGLT2 inhibitors were derived from the meta-analysis. We used time-varying risk ratios for all-cause death and hospitalization for heart failure, time-invariant risk ratio for genital infection.

^d^ All costs were expressed in US dollars ($), inflation-adjusted to 2022 according to the Consumer Price Index for medical service (as detailed in Supplementary Appendix SA1).

^e^ We derived the cost of genital infection per event based on clinical experience.

Abbreviations: BUN: blood urea nitrogen; eGFR: estimated glomerular rate filtration; HF: heart failure; HFrEF: heart failure and reduced ejection fraction; NA: not applicable; NT-proBNP: N-terminal pro-B-type natriuretic peptide; NYHA: new york heart association; QALY: quality-adjusted life year; SBP: systolic blood pressure; SGLT2: sodium-glucose cotransporter-2.

### 2.6 Costs and quality-adjusted life years

Data on direct medical costs for SGLT2 inhibitors, standard therapy, hospitalization for heart failure, and genital infection were taken from published literature and clinical experience in the present study (Table 2) (Huang et al., 2017; Sichuan Pharmaceutical Equipment Bidding and Purchasing Service Center, 2024; National Health Commission of the People’s Republic of China, 2022). All costs were adjusted to 2022 inflation levels according to the Consumer Price Index (CPI) for medical services and converted to US dollars at the 2022 annual average exchange rate of 1$ = 6.7261 CNY (National Bureau of Statistics of China, 2023; China Foreign Exchange Trade System, 2024).

We measured health outcomes under two treatment strategies with QALYs. The calculation of QALYs assigned different utility values to specific health states and incorporated the time spent in those states. The baseline utility values of patients under different New York Heart Association (NYHA) classifications and event-specific disutility values were obtained from the extant published literature (Göhler et al., 2009; Barry et al., 1997). The model population experiencing hospitalization for heart failure or genital infection demonstrated a short-term decrease in health utility (as detailed in Supplementary Appendix SA1).

### 2.7 Statistical analysis

#### 2.7.1 Base case analysis

The base case analysis estimated overall all-cause deaths, total events of hospitalizations for heart failure, average life years per person, average cost per person, and average QALYs per person in 2 years under two treatment strategies separately. The primary outcome of the microsimulation model was the ICER of adding SGLT2 inhibitors to standard therapy versus standard therapy alone. Considering statistical uncertainty in a single simulation, the simulations were reiterated 10,000 times, and the distribution of results was visualized with a scatter plot. The average ICER when SGLT2 inhibitors were found to be more effective was determined. We applied an annual discount rate of 3% to QALYs and costs and tested a range of 0%–8% in sensitivity analysis. One to three times the Gross Domestic Product (GDP) *per capita* in China in 2022 ($12,741) was set as the willingness-to-pay (WTP) threshold according to the recommendations of China Guidelines for Pharmacoeconomic Evaluations 2020 (Chinese Pharmaceutical Association, 2020). SGLT2 inhibitors were considered cost-effective if the ICER was less than three times the GDP *per capita* and highly cost-effective when less than the GDP *per capita*. We plotted the cost-effectiveness acceptability curve based on the results of probabilistic sensitivity analysis and revised the manuscript accordingly.

#### 2.7.2 Scenario and subgroup analyses

To examine the trend of cost-effectiveness over time, we simulated the patient trajectories under two treatment strategies in 0.5, 1, and 1.5 years. Subgroup analyses were conducted by dividing the model population based on age, sex, diabetes, and chronic kidney disease.

#### 2.7.3 Sensitivity analyses

Uncertainty in model inputs was minimized through one-direction and probabilistic sensitivity analyses. In one-direction sensitivity analysis, the impact of model inputs on ICER was assessed by varying a chosen parameter within a specific range while keeping all other parameters constant. The risk ratio parameters varied within ±20% of the original value; the utility values varied within ±10% of the original value, and the transition probabilities and costs varied from 1/3 to 3 times the original value. To perform probabilistic sensitivity analysis, we ran the microsimulation model 10,000 times using Monte Carlo simulation, with multiple parameters randomly sampled according to corresponding probability distribution simultaneously each time (Table 2). Based on results of probabilistic sensitivity analysis, we plotted the cost-effectiveness acceptability curve (CEAC) to address the uncertainty of WTP threshold (as detailed in Supplementary Appendix SA1).

## 3 Results

### 3.1 Model population

The current simulation study included 4,227 patients with HFrEF who were discharged between 1 January 2011 and 30 September 2018. The model population showed a median age of 63 [interquartile range (IQR), 52 to 72] years old; 1,132 (26.8%) were women, 942 (22.3%) had diabetes, and 2,797 (66.2%) were in NYHA class III to IV. The mean and median of LVEF were 31.9% (standard deviation [SD], 6.25) and 33% (IQR, 28–37), respectively (Table 1). The median predicted risks for all-cause death and hospitalization for heart failure under standard therapy alone in the first 6 months were 9.2% (IQR, 5.2%–15.4%) and 15.9% (IQR, 12.6%–20.1%), respectively (Supplementary Appendix SA3), which corroborated findings of earlier epidemiological studies (Huang et al., 2021; Lawson et al., 2019). When adding SGLT2 inhibitors, the risks were reduced to 8% (IQR, 4.5%–13.4%) for all-cause death and 9.7% (IQR, 7.7%–12.3%) for hospitalization for heart failure in the first cycle.

### 3.2 Base case analysis

During the 2-year simulation, giving SGLT2 inhibitors and standard treatment resulted in 1,336 (31.6%) deaths and 1,877 re-hospitalizations; it implied 32 fewer deaths and 228 fewer hospitalizations compared to the treatment without using SGLT2 inhibitors (Table 3). Patients had, on average, 1.578 life years and 1.047 QALYs when adding SGLT2 inhibitors to standard therapy, 1.528 life years, and 1.014 QALYs when receiving standard therapy alone. The average cost per person was $9,045.42 with SGLT2 inhibitors plus standard therapy and $8,744.69 with standard therapy alone, thereby yielding an average ICER of $9,355.66 per QALY gained, which was considered highly cost-effective. Adding SGLT2 inhibitors was found cost-effective for 99.94% of the simulations and highly cost-effective for 99.19% (Supplementary Appendix SA4). We plotted the cost-effectiveness acceptability curve based on the results of probabilistic sensitivity analysis and revised the manuscript accordingly.

**TABLE 3:** Results of base case analysis and scenario analysis in different time horizons.

	Overall all-cause deaths	Overall hospitalizations for heart failure	Average life years per person	Incremental life years per person	Average cost per person ($)	Incremental cost per person ($)	Average QALYs per person	Incremental QALYs per person	Average ICER, $/QALY
Base case analysis (2-year time horizon)									
SGLT2 inhibitors plus standard therapy	1,336	1,877	1.578	0.049	9,045.42	300.74	1.047	0.033	9,355.66
Standard therapy alone	1,471	2,840	1.528	NA	8,744.69	NA	1.014	NA	NA
Scenario analyses (1.5-year time horizon)									
SGLT2 inhibitors plus standard therapy	1,068	1,407	1.236	0.033	7,111.14	204.93	0.825	0.022	9,350.82
Standard therapy alone	1,190	2,178	1.202	NA	6,906.21	NA	0.803	NA	NA
Scenario analyses (1-year time horizon)									
SGLT2 inhibitors plus standard therapy	760	927	0.862	0.019	4,977.11	115.38	0.579	0.013	9,241.80
Standard therapy alone	859	1,484	0.843	NA	4,861.73	NA	0.567	NA	NA
Scenario analyses (0.5-year time horizon)									
SGLT2 inhibitors plus standard therapy	407	434	0.452	0.007	2,610.35	51.83	0.306	0.005	12,346.07
Standard therapy alone	468	711	0.445	NA	2,558.53	NA	0.301	NA	NA

Abbreviations: ICER: incremental cost-effectiveness ratio; NA: not applicable; QALYs: quality-adjusted life years; SGLT2: sodium-glucose cotransporter-2.

### 3.3 Time-varying cost-effectiveness analyses

Table 3 summarizes the simulations in different timeframes. With the extension of treatment, the average ICER decreased from $12,346.07 per QALY gained at 0.5 years to $9,355.66 per QALY gained at 2 years. Distributions of results were visualized with scatter plots (Supplementary Appendix SA5). As treatment continued, more simulated results were in the area where SGLT2 inhibitors plus standard therapy was more effective (98.63% at 0.5 years, 99.79% at 1 year, 99.94% at 1.5 years, and 99.96% at 2 years), cost-effective (97.93% at 0.5 years, 99.70% at 1 year, 99.92% at 1.5 years, and 99.94% at 2 years), and highly cost-effective (77.37% at 0.5 years, 96.76% at 1 year, 98.48% at 1.5 years, and 99.19% at 2 years).

### 3.4 Subgroup analyses

We separated the study population into following subgroups based on different baseline characteristics: 1) 1,132 women and 3,095 men; 2) 2,238 patients ≥63 years of age (the median age of study population) and 1,989 patients <63 years old; 3) 942 patients with diabetes and 3,285 patients without diabetes; 4) 3,289 patients with an eGFR ≥60 mL/min/1.73 m^2^ and 938 patients with an eGFR <60 mL/min/1.73 m^2^ (Supplementary Appendix SA6). Subgroup simulations yielded an average ICER less than three times the GDP *per capita* in the female subgroup and subgroup with declined kidney function (eGFR <60 mL/min/1.73 m^2^), an average ICER less than the GDP *per capita* in other subgroups. The results confirmed the cost-effectiveness of adding SGLT2 inhibitors across heterogeneous populations (Supplementary Appendices SA7, SA8).

### 3.5 Sensitivity analyses

**FIGURE 2:**
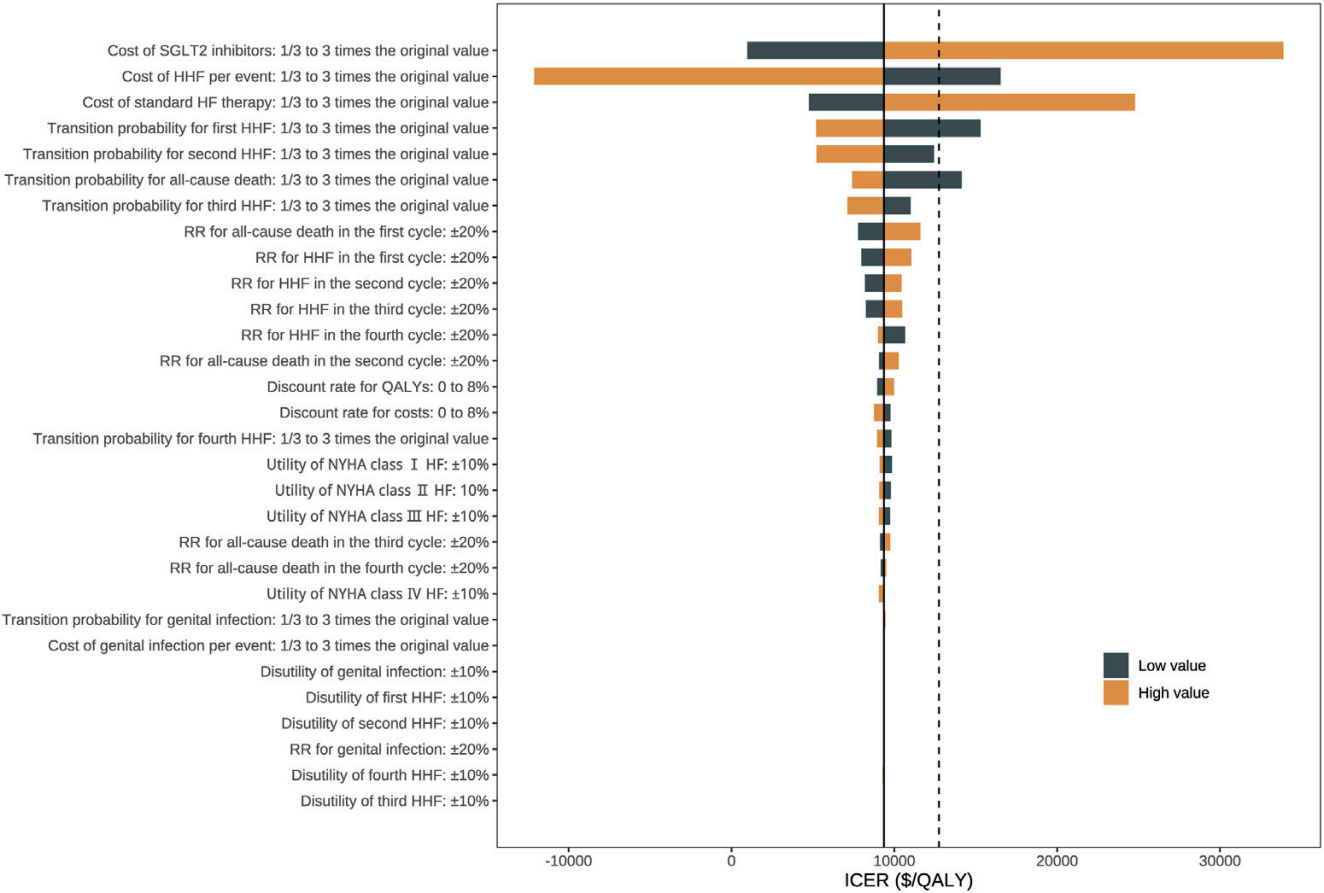
Tornado diagram for one‐direction sensitivity analysis. Notes: The black solid line represents the average ICER in base case analysis, and the black dashed line represents the willingness‐to‐pay threshold of GDP per capita. Abbreviations: HF: heart failure; HHF: hospitalization for heart failure; ICER: incremental cost‐effectiveness ratio; NYHA: New York Heart Association; QALY: quality-adjusted life year; RR: risk ratio; SGLT2: sodium-glucose cotransporter‐2.

**FIGURE 3:**
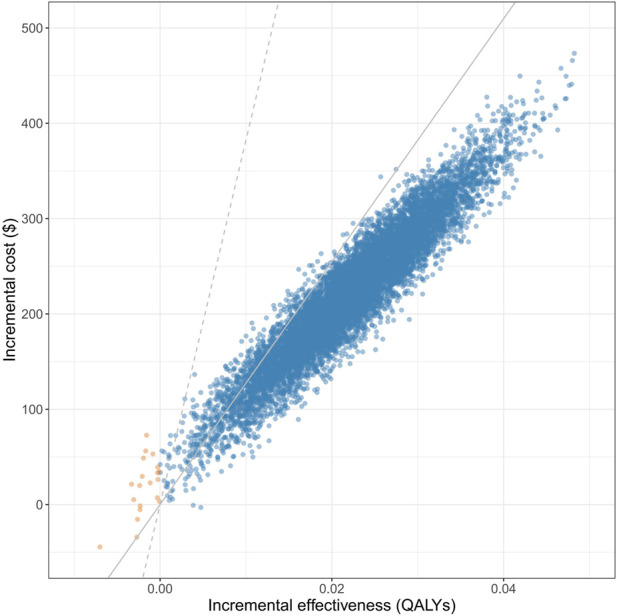
Cost-effectiveness plane for probabilistic sensitivity analysis. Notes: Figure 3 shows the distribution of 10,000 results in the probabilistic sensitivity analysis. The gray solid line represents the willingness-to-pay threshold of GDP per capita, and the gray dashed line represents the willingness-to-pay threshold of three times GDP per capita. Abbreviations: QALYs: quality-adjusted life years; SGLT2: sodium-glucose cotransporter-2.

The one-direction sensitivity analysis indicated that the ICER was highly influenced by the cost of SGLT2 inhibitors, the cost of hospitalization for heart failure, the cost of standard therapy, and the baseline risks of all-cause death and hospitalization for heart failure (Figure 2). When switching the cost of SGLT2 inhibitors from one-third of its base value to three folds, the ICER ranged from $ 970.7 per QALY gained to $ 33,893.6 per QALY gained, which remains less than the WTP threshold of three times GDP *per capita*. When the cost of hospitalization for heart failure trebled, the ICER of adding SGLT2 inhibitors was –$12,112.7 per QALY gained, indicating dominant cost-effectiveness. For patients at higher baseline risks of adverse events, SGLT2 inhibitors showed more pronounced cost-effectiveness. The cost-effectiveness showed robustness when changing other parameters (Supplementary Appendix SA9). Among 10,000 iterations, adding SGLT2 inhibitors was more effective in 99.80% of simulations, cost-effective in 99.67% of simulations, and highly cost-effective in 94.71% of simulations (Figure 3). The CEAC demonstrated visually that, compared with standard therapy alone, SGLT2 inhibitors plus standard therapy were not cost-effective when the WTP threshold was less than around $7,500; of similar cost-effectiveness when the WTP threshold was around $10,000; highly cost-effective when the WTP threshold was around $15,000 or higher (Supplementary Appendix SA10).

## 4 Discussion

By constructing a microsimulation model with real-world patient-level data, the current study demonstrated that SGLT2 inhibitors were a highly cost-effective add-on to standard therapy for people with HFrEF in China. In the study population, adding SGLT2 inhibitors for 2 years prevented 32 deaths and 228 hospitalizations for heart failure, and increased 49 life years for every 1,000 patients treated, and showed an enhanced cost-effectiveness over time. Consistency was observed in results among different subgroups. To our knowledge, the present study is first of its kind that adopted a microsimulation model to assess the cost-effectiveness of SGLT2 inhibitors in people with HFrEF from the perspective of the Chinese healthcare system. The simulation estimating both the time-varying effectiveness and cost-effectiveness of adding SGLT2 inhibitors provided new insights into health policymaking.

There were some limitations noticed in the present study. First, the study population was recruited in a single medical center in China, which could be less representative of the country. Nevertheless, the individual-level data allowed precise subgroup estimation, which was more useful for individual-level decision-making. Subgroup analyses according to major prognostic factors and sensitivity analyses with varying model parameters proved the robustness of cost-effectiveness. Simulating target heart failure populations in different regions or clinical settings with the model and input parameters adapted to local contexts can provide key insights into the broader use of SGLT2 inhibitors. Second, the current evidence for SGLT2 inhibitors is unavailable after 2 years of treatment, which is essential for time-varying estimation and associated decision-making. Modeling updates are on schedule following the release of longer-term randomized trials and well-designed real-world studies that could inform clinical practice. Third, emerging evidence established that SGLT2 inhibitors reduce worsening heart failure and cardiovascular death in people with heart failure and preserved ejection fraction (HFpEF). Compared with HFrEF, HFpEF accounts for a higher proportion of Chinese patients with heart failure but has limited therapy (Chen et al., 2022). The cost-effectiveness analysis of SGLT2 inhibitors in people with HFpEF is warranted to contribute to incorporating SGLT2 inhibitors into treatment for Chinese patients with HFpEF. With the novel concept of cardiovascular-kidney-metabolic (CKM) syndrome, the potential implications of implementing SGLT2 inhibitors among people with diverse CKM health conditions are worth investigating (Ndumele et al., 2023; Pan et al., 2024; Wang and Li, 2024). Fourth, without a locally validated risk calculator or localized input parameter (e.g., incidence rate of genital infection and utility values) from the target Chinese population, this microsimulation adopted a prediction model and parameters from other populations (Voors et al., 2017). It led to potential indirectness for the evidence implementation. Nevertheless, previous studies suggested the consistency of risk factors in Chinese and Western patients with heart failure (Zhang et al., 2017; Yang et al., 2020). The sensitivity analyses also confirmed that this indirectness may not be important in interpretation.

As the cost of hospitalization for heart failure accounts for approximately 66% of the total medical costs of heart failure (Huang et al., 2017), the prevention of recurrent hospitalizations for heart failure is the economic cornerstone for the treatment of heart failure. Although previous evidence synthesis confirmed the diminishing relative effects of SGLT2 inhibitors in patients with heart failure over time (Zou et al., 2024; Zou et al., 2022), the results of this microsimulation study using real-world patients in China indicate an enhanced cost-effectiveness over time. The inconsistency of the trends in effectiveness and cost-effectiveness could be due to our model considering the shift of patient characteristics over time, which enhanced the applicability of the current study in real-world practice and policymaking. This model resulted in an increasing incidence of hospitalization episodes and dynamic baseline risks over time, which neutralized the diminishing relative effects of SGLT2 inhibitors. The finding was consistent with previous studies based on lifetime Markov models that yielded an ICER of $1,893.59 to $6,946.69 per QALY gained in 10- to 20-year time horizons (Jiang et al., 2022; Lin et al., 2022). Previous studies revealed that the prognosis of heart failure varied according to age, sex, diabetes status, and kidney function (Wang et al., 2024; Zhang et al., 2017). The present study incorporating individual prognostic factors and conducting subgroup analysis confirmed consistent cost-effectiveness of SGLT2 inhibitors across heterogeneous populations. The wide use of SGLT2 inhibitors in the eligible but heterogeneous heart failure population is encouraged.

The cost of SGLT2 inhibitors, at the population level, is the most important factor for the cost-effectiveness. The price negotiation of SGLT2 inhibitors is thus critical in the healthcare system, although the current price of SGLT2 inhibitors in China reaches the WTP threshold in over 99% of the simulations. The current price of SGLT2 inhibitors in China allows their wider application in patients with heart failure for better public health service and calls for further optimization in real-world practice, thereby supporting the authority of medication initiation in primary care. Our study suggested patients with longer life expectancy, newly diagnosed heart failure, and good adherence could be the best responders to SGLT2 inhibitors, which showed a step-by-step implementation in a wider healthcare system.

## 5 Conclusion

Adding SGLT2 inhibitors to standard therapy was found to be highly cost-effective for Chinese patients with HFrEF, and the cost-effectiveness appeared to enhance over time. The cost of SGLT2 inhibitors, the cost of hospitalization for heart failure, the cost of standard therapy, and baseline risks of all-cause death and hospitalization for heart failure were the most critical factors for the cost-effectiveness of the medication. In the future, longer-term randomized trials and well-designed real-world evidence are warranted to further trigger the update of this microsimulation.

## Data Availability

The original contributions presented in the study are included in the article/Supplementary Material. Further inquiries can be directed to the corresponding author.
