# Supplementary material for: Time-varying cost-effectiveness analysis of sodium-glucose cotransporter-2 inhibitors in Chinese patients with heart failure and reduced ejection fraction: A microsimulation of the real-world population
Source: Front Pharmacol. 2025 Feb 25;16:1527972. doi: 10.3389/fphar.2025.1527972 (PMC11893398; doi:10.3389/fphar.2025.1527972)
Supplement: Supplementary file 1 [file DataSheet1.pdf]

Supplementary materials

Time-varying cost-effectiveness analysis of sodium-glucose cotransporter-2 inhibitors in Chinese patients with heart failure and reduced ejection fraction: a microsimulation of real-world population

Table of contents

Appendix 1. Supplement to the Method section..2
Appendix 2. Flow diagram for patient selection 5
Appendix 3. Baseline risks of model population..6
Appendix 4. Simulation results of base case analysis 7
Appendix 5. Simulation results for scenario analyses..8
Appendix 6. Baseline characteristics of subgroup population 9
Appendix 7. Results of subgroup analyses..17
Appendix 8. Simulation results for subgroup analyses 18
Appendix 9. Results of one-direction sensitivity analysis..20
Appendix 10. Cost-effectiveness acceptability curve of the probabilistic sensitivity analysis 22
References 23

Appendix 1. Supplement to the Method section

Model population selection

Eligible patients were those who 1) were discharged between January 1, 2011 and September 30, 2018; 2) had a length of stay >2 days; 3) were ≥18 years old and <90 years old; 4) had documented echocardiographic evidence of a left ventricular ejection fraction (LVEF) ≤40%; 5) had complete records of relevant prognostic factors (i.e., age, systolic blood pressure [SBP], serum creatinine, hemoglobin, blood urea nitrogen [BUN], and N-terminal pro-B-type natriuretic peptide [NT-proBNP]), diagnoses at discharge, and prescription. We identified patient status at discharge in the electronic medical records system and excluded those with unplanned discharge or in-hospital death. During patient selection, due to the restricted use of SGLT2 inhibitors in people with declined kidney function, we excluded patients with an estimated glomerular rate filtration (eGFR) <30 mL/min/1.73m^2 as calculated by the chronic kidney disease epidemiology collaboration formula using age, sex, race, and serum creatinine values.[1]

Data collection

When there were multiple hospitalization records of one patient meeting the eligibility criteria, we extracted information from the first hospitalization. We collected following data for every patient: 1) age, sex, smoking and alcohol consumption status, admission and discharge dates and times, physical examination at admission, prescription, discharging diagnoses with International Classification of Diseases, 10th Revision (ICD-10 codes) and other diagnosis terms, and patient status from the electronic medical record system; 2) vital signs (SBP, diastolic blood pressure [DBP], and heart rate), height, and weight from the nursing system; 3) laboratory test results from the laboratory information system (LIS); 4) LVEF from the echocardiography reading. Outliers in SBP (>200mm Hg or <80mm Hg) and hemoglobin (<60g/L or >200g/L) were identified based on clinical experience and considered as missing values. The New York Heart Association (NYHA) classification of patients was derived from information including diagnoses, with missing values imputed based on age, sex, LVEF, NT-proBNP, and eGFR via predictive mean matching method. Patients were deemed to have peripheral edema at baseline if lower extremity or facial edema was identified in their physical examination at admission. Comorbidities (e.g., hypertension, diabetes mellitus, and acute myocardial infarction) were identified using the discharging ICD-10 codes (I10-I15 for hypertension; E10-E14 for diabetes mellitus; I144-I49 for arrhythmia; I20-I25 for ischemic heart disease; I42 for cardiomyopathy; I01-I09 for rheumatic heart disease; I21-I23 for acute myocardial infarction; J40-J44 for chronic obstructive pulmonary disease) and other diagnosis terms.

Model input parameters

Transition probabilities

Transition probabilities for all-cause death and hospitalization for heart failure for patients under standard therapy alone in the first cycle (i.e., baseline risk) were predicted by the BIOSTAT-CHF prediction model based on individual baseline characteristics.[2] The derivation cohort of the BIOSTAT-CHF prediction model included 2,516 European people with heart failure and reduced or preserved ejection fraction, the mean age was 68.9 years old, 26.6% were female, and 83.3% were in NYHA II-III (Table S1). The compact prediction model predicted risk for all-cause death with age, BUN, hemoglobin, NT-proBNP, and beta-blocker use, and predicted the risk for hospitalization for heart failure with age, SBP, eGFR, presence of peripheral edema, and history of hospitalization for heart failure in the past year, yielding c-

statistic values of 0.69 and 0.67 respectively. The mean values of regression coefficients and corresponding standard errors for predictive variables were converted from reported hazard ratios and 95% confidence interval. The regression coefficients had normal distribution. The prediction equation was as follows:

$$Transition\ probability = 1 - \lambda^{e^{[\sum_{i=1}^{p}\beta_i X_i - mean(\sum_{i=1}^{p}\beta_i X_i)]}}$$

$\sum_{i=1}^{p}\beta_i X_i$ represents the sum of the products of each predictive variable and its regression coefficient. We adjusted the prediction equation with the risk of all-cause death and hospitalization for heart failure in Chinese heart failure population. The risks in Chinese heart failure population were extracted from the China PEACE 5p-HF study, which enrolled 4,866 patients hospitalized due to heart failure from 52 hospitals across 37 cities in China between August 2016 to May 2018 (Table S1).[3] λ represents the incidence of all-cause death or hospitalization for heart failure in 6 months in Chinese heart failure population. λ equals to 0.0875 when predicting the risk of all-cause death, 0.1595 for hospitalization for heart failure. We assumed that all patients had a history of hospitalization for heart failure in the past year because the study population were derived from hospitalization records.

Table S1. Comparison between the BIOSTAT-CHF and China PEACE 5p-HF cohorts

Baseline characteristics	BIOSTAT-CHF	China PEACE 5p-HF
Sample size, n	2,516	4,866
Age, years	68.9±12	65.2±13.5
Female, n (%)	670 (26.6)	1843 (37.6)
Systolic blood pressure, mmHg	124.7±21.9	133.2±24.7
Heart failure with reduced ejection fraction, n (%)	2,354 (93.6)	1,850 (37.8)
New York Heart Association II-III, n (%)	2,096 (83.3)	2,868 (58.9)
New-onset heart failure	702 (27.9)	1,440 (29.4)
Diabetes, n (%)	819 (32.6)	1,539 (31.4)

We derived the transition probabilities for genital infection per cycle from a UK cohort study.[4] The cohort study included 125,237 females and 146,603 males in the primary care setting from the UK General Practice Research Database (GPRD). All patients were followed for 1 year from their study index date for the first record of an infection or a censored event. Among the female patients without diabetes (n= 62,700; median age 62 years; 57,844 PY of follow-up), the incidence of vaginitis was 10.3/1,000PY (95% CI 9.5 to 11.1), with the risk being higher in those with diabetes (adjusted RR 1.81, 95% CI 1.64 to 2.00), with prior genital tract infection (adjusted RR 17.75, 95% CI 13.54 to 23.27). For female patients with diabetes, the risk was 6.99 (95% CI 5.97 to 8.18) greater in those with prior genital infection. The overall incidence of balanitis was 2.5/1,000PY (95% CI 2.1 to 2.9) for patients without diabetes, with the risk being higher in those with diabetes (adjusted RR 2.85, 95% CI 2.39 to 3.39), with prior genital tract infection (adjusted RR 62.80, 95% CI 37.38 to 105.50). For male patients with diabetes, the risk was 11.22 (95% CI 9.02 to 13.97) greater in those with prior genital infection. We converted the incidence rate to probability with following equation: $Probability = 1 - exp(-rate * t)$, where t represents the timeframe (that is, 0.5 here). The transition probabilities had log-normal distribution.

Costs

The prices of SGLT2 inhibitors approved for heart failure in China during the model development were

$0.65 for dapagliflozin (Forxiga) 10 mg per tablet and $0.63 for empagliflozin (JARDIANCE) 10 mg per tablet.[5] Therefore, the cost of SGLT2 inhibitors per cycle was the average cost of dapagliflozin and empagliflozin in 6 months ($116.67). We extracted the average annual cost of standard therapy among patients with heart failure who were free of hospitalization for heart failure from a real-world study.[6] The study examined treatment costs associated with heart failure in China using data from the 2014 national insurance database sponsored by the China Health Insurance Research Association (CHIRA), that covers national urban employees and residents. The medical expenditure of hospitalization for heart failure per event was 9,326 CNY ($1,386.54) in Chinese public hospitals in 2021 according to 2022 China Health Statistics Yearbook.[7] The cost of genital infection per event consisting of outpatient visits, laboratory tests, and antifungal agents was calculated based on data from a tertiary hospital. All costs were inflation-adjusted to 2022 according to the Consumer Price Index (CPI) for medical services.[8] The CPIs from 2015 to 2022 were 102.7, 103.8, 106, 104.3, 102.4, 101.8, 100.4, and 100.6 respectively. For example, the annual cost of standard heart failure therapy derived from the 2014 database was inflation-adjusted to 2022 by multiplying the original value ($4,281.08) by CPIs from 2015 to 2022. The equation is as follows:

$$inflation - adjusted\ cost = 4281.08 \times 102.7\% \times 103.8\% \times 106\% \times 104.3\% \times 102.4\% \times 101.8\% \times 100.4\% \times 100.6\% = 5{,}312.41$$

Quality-adjusted life years

Utilities of heart failure under different NYHA classifications and disutilities regarding hospitalization for heart failure were derived from the Eplerenone Post-acute Myocardial Infarction Heart Failure Efficacy and Survival Study trial,[9] which indirectly measured health status of 1,395 patients with chronic heart failure after acute myocardial infarction using EuroQol (EQ-5D) and then converted the EQ-5D health states into utility weights via preference-based scoring algorithms. The utilities of patients with stable heart failure with NYHA classification I to IV are 0.855, 0.771, 0.673, and 0.532, respectively. Utilities of patients who experienced hospitalization for heart failure or genital infection were the sum of initial utility and event-specific disutility. Disutilities when hospitalized for heart failure varied with the number of hospitalizations. Patients with more rehospitalizations had greater impairment of health.[9] The disutility of genital infection was -0.003.[10] Duration of the hospitalization condition needed for the calculation of QALYs was 11.23 days, which was consistent with the average length of hospital stay among model population. The duration of genital infection was randomly sampled from 7 to 14 days because genital infection associated with SGLT2 inhibitors was mostly of mild intensity and can be cured with antifungal agents within 1 to 2 weeks.

Statistical analyses

Cost-effectiveness acceptability curve (CEAC)

Based on 10,000 simulated results in the probabilistic sensitivity analysis, we calculated net monetary benefit (NMB) under different willingness-to-pay (WTP) thresholds. The equation is as follows:

$$NMB = \Delta QALYs * WTP\ threshold - \Delta Costs$$

When NMB >0, SGLT2 inhibitors plus standard therapy was cost-effective compared with standard therapy alone under the given WTP threshold. The proportions that SGLT2 inhibitors plus standard therapy was cost-effective in 10,000 simulations under different WTP thresholds were calculated. The x-axis of the CEAC represents various WTP thresholds while the y-axis is the proportion of being cost-effective.

Appendix 2. Flow diagram for patient selection

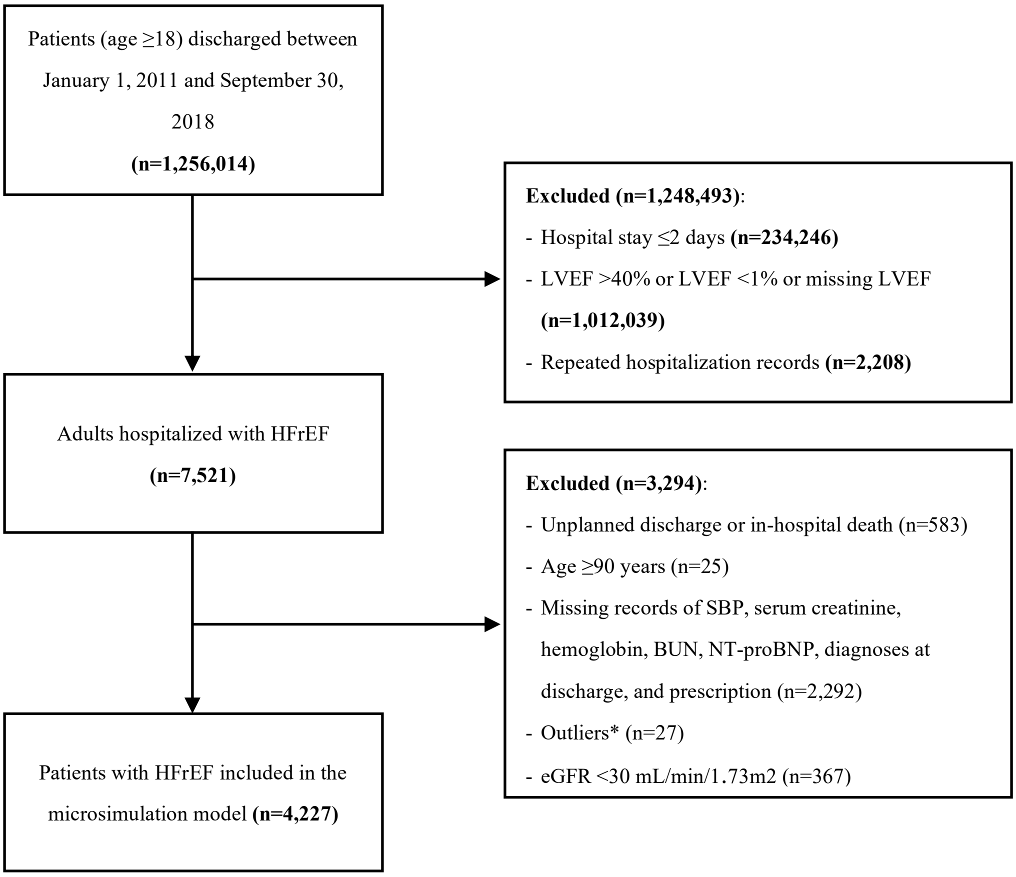

* Outliers refer to systolic blood pressure >200mm Hg or <80mm Hg, and hemoglobin <60g/L or >200g/L.

BUN: blood urea nitrogen; eGFR: estimated glomerular rate filtration; HFrEF: heart failure with reduced ejection fraction; LVEF: left ventricular ejection fraction; NT-proBNP: N-terminal pro-B-type natriuretic peptide; SBP: systolic blood pressure.

Appendix 3. Baseline risks of model population

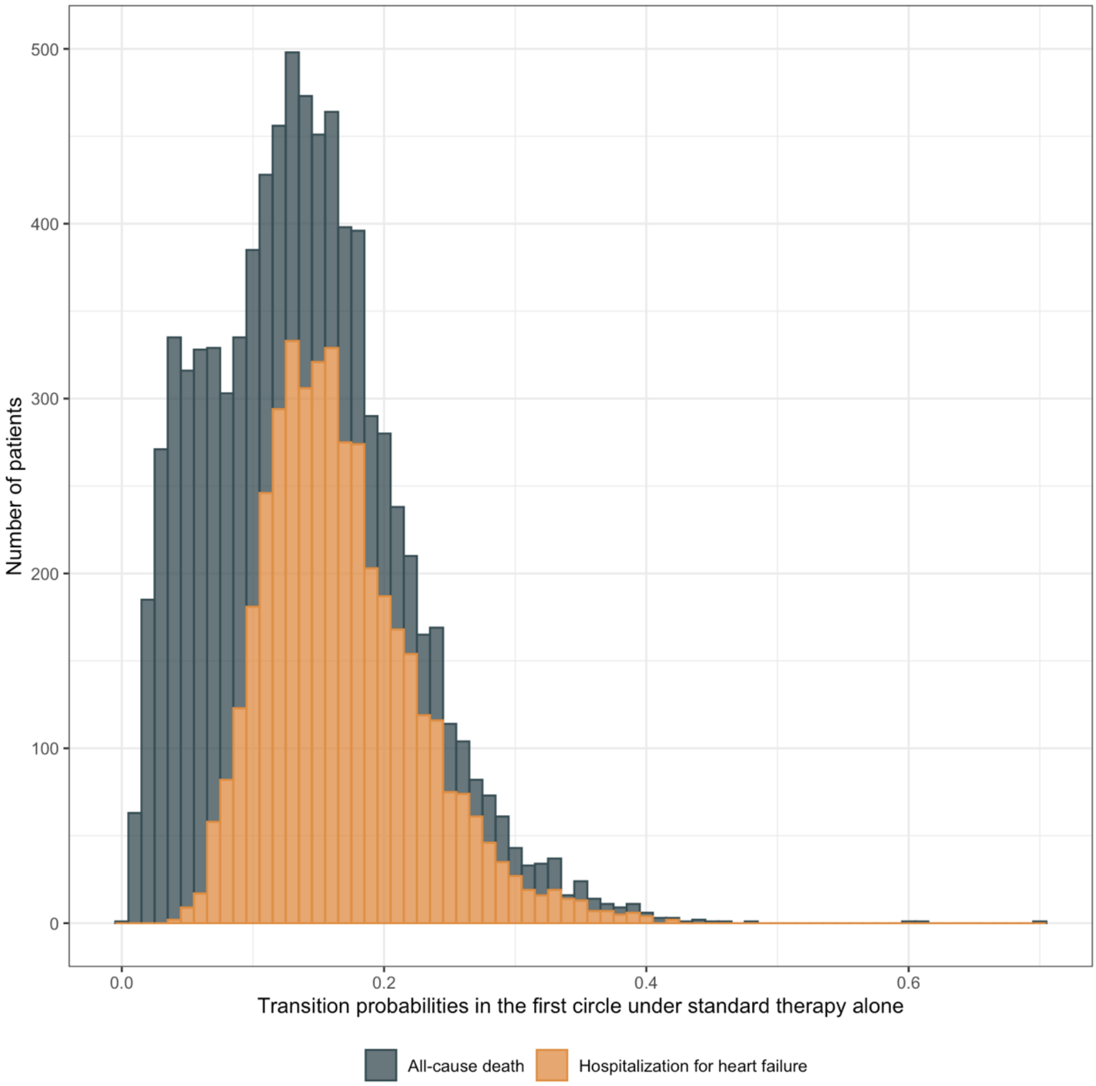

Appendix 4. Simulation results of base case analysis

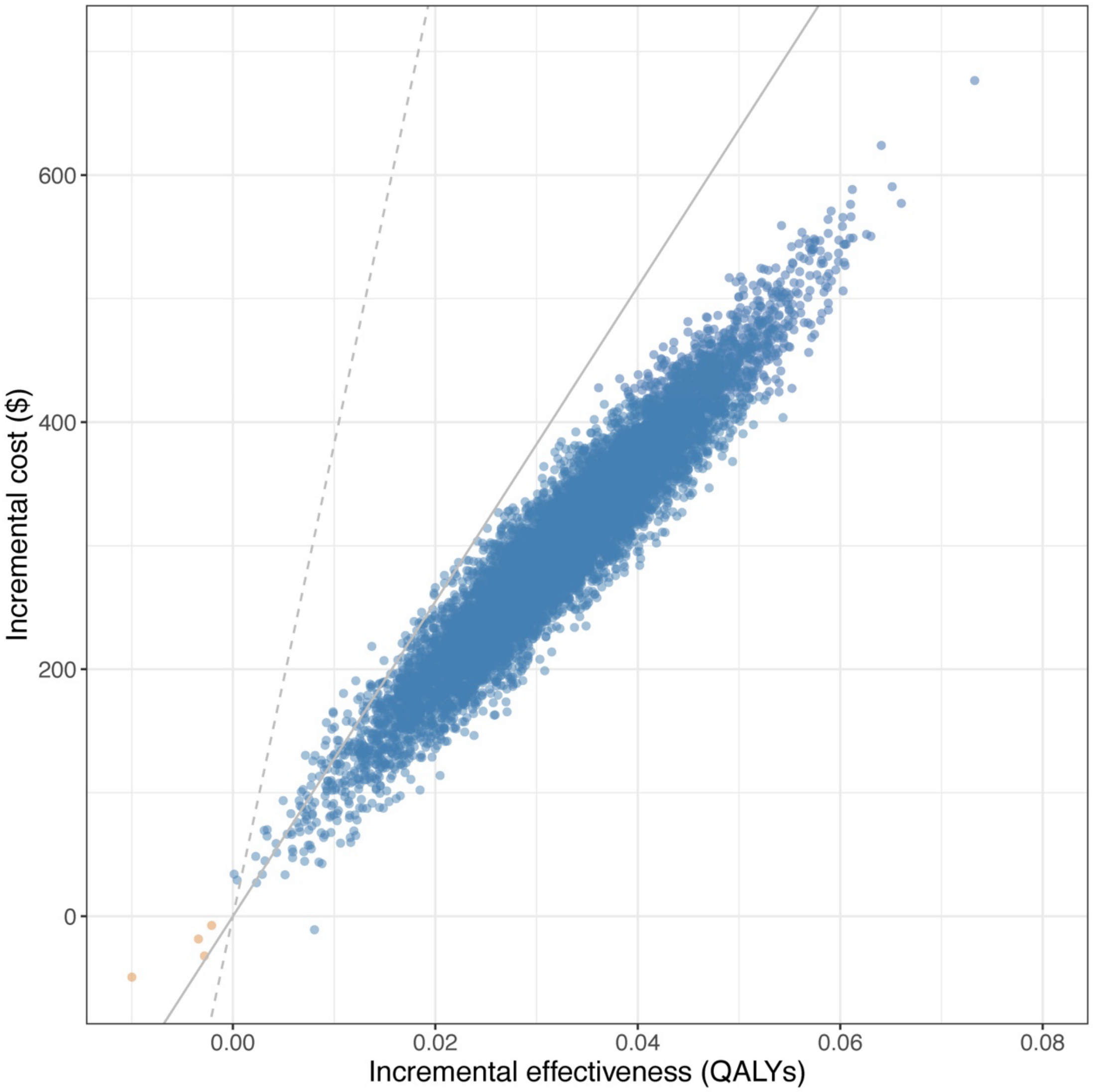

Appendix 4 shows the distribution of 10,000 results in the base case analysis. The x-axis represents incremental QALYs per person between SGLT2 inhibitors plus standard therapy and standard therapy alone, and the y-axis represents incremental cost per person. The grey solid line represents the willingness-to-pay threshold of GDP per capita ($12,741/QALY), while the grey dashed line represents the willingness-to-pay threshold of three times GDP per capita ($38,223/QALY). The orange dots represent that SGLT2 inhibitors plus standard therapy were not more effective than standard therapy alone (incremental QALYs <0), the blue dots represent that SGLT2 inhibitors plus standard therapy were more effective than standard therapy alone (incremental QALYs >0).

QALYs: quality-adjusted life years; SGLT2: sodium-glucose cotransporter-2.

Appendix 5. Simulation results for scenario analyses

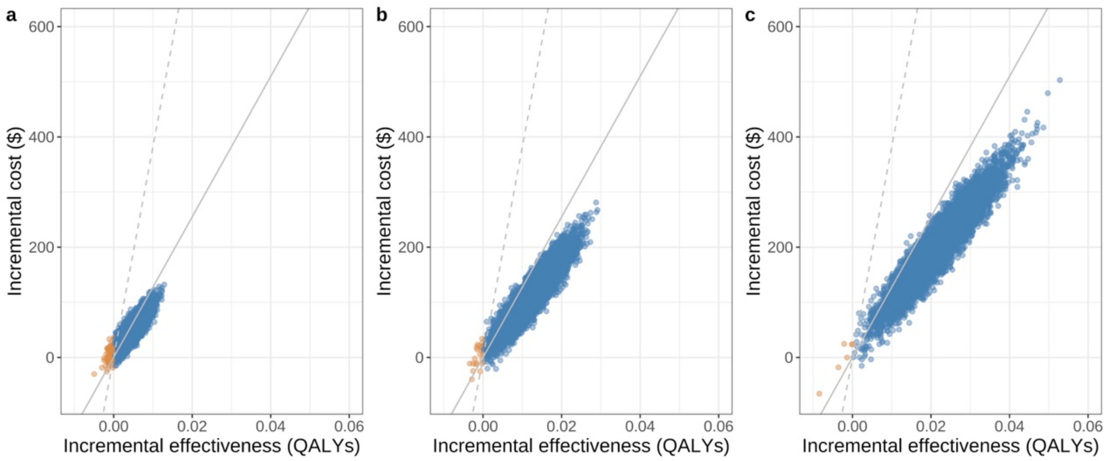

Appendix 5a-c shows the distribution of 10,000 results in the scenario analyses at 0.5, 1, and 1.5 years, respectively. The x-axis represents incremental QALYs per person between SGLT2 inhibitors plus standard therapy and standard therapy alone, and the y-axis represents incremental cost per person. The grey solid line represents the willingness-to-pay threshold of GDP per capita ($12,741/QALY), while the grey dashed line represents the willingness-to-pay threshold of three times GDP per capita ($38,223/QALY). The orange dots represent that SGLT2 inhibitors plus standard therapy were not more effective than standard therapy alone (incremental QALYs <0), the blue dots represent that SGLT2 inhibitors plus standard therapy were more effective than standard therapy alone (incremental QALYs >0).

QALYs: quality-adjusted life years; SGLT2: sodium-glucose cotransporter-2.

Appendix 6. Baseline characteristics of subgroup population

Appendix 6A. Baseline characteristics of subgroup population according to sex

	Females (n = 1,132)	Males (n = 3,095)
Age, median (IQR), years	64 (53, 73)	63 (52, 72)
Heart rate, median (IQR), beats/minute	76 (70, 84)	76 (70, 84)
SBP, median (IQR), mm Hg	119 (107, 130)	120 (108, 131)
DBP, median (IQR), mm Hg	67 (60, 75)	69 (61, 78)
Alcohol use, No. (%)	70 (6.2)	1582 (51.1)
Smoking, No. (%)		
No	1,100 (97.2)	1,105 (35.7)
Current	16 (1.4)	748 (24.2)
Past	16 (1.4)	1,242 (40.1)
Peripheral edema, No. (%)	185 (16.3)	428 (13.8)
LVEF, median (IQR), %	33 (28, 37)	33 (28, 37)
Distribution, No. (%)		
30 to 40%	792 (70.0)	2,072 (66.9)
20 to 29%	303 (26.7)	916 (29.6)
≤19%	37 (3.3)	107 (3.5)
NYHA classification, No. (%)		
I	162 (14.3)	528 (17.1)
II	172 (15.2)	568 (18.4)
III	507 (44.8)	1,285 (41.5)
IV	291 (25.7)	714 (23.1)
eGFR, median (IQR), mL/min/1.73 m²	78.45 (59.86, 96.17)	80.37 (63.28, 95.00)
BUN, median (IQR), mmol/L	6.59 (5.02, 8.58)	7.10 (5.56, 9.10)
Hemoglobin, median (IQR), g/L	122 (108, 135)	136 (121, 149)
NT-proBNP, median (IQR), pg/mL	21,153 (2,102, 35,000)	14,209 (1,937, 35,000)
HbA1c, median (IQR), %	5.29 (4.72, 6.12)	5.29 (4.72, 6.30)
Medical history, No. (%)		
Diabetes mellitus	228 (20.1)	714 (23.1)
Chronic obstructive pulmonary disease	130 (11.5)	593 (19.2)
Hypertension	374 (33.0)	1,167 (37.7)
Acute myocardial infarction	132 (11.7)	493 (15.9)
Ischemic heart disease	401 (35.4)	1,553 (50.2)
Rheumatic heart disease	391 (9.3)	391 (9.3)
Cardiomyopathy	345 (30.5)	879 (28.4)
Arrhythmia	487 (43.0)	1,190 (38.4)
Medical treatment, No. (%)		
Diuretics†	902 (70.8)	2,109 (68.1)

Beta-blockers	782 (69.1)	2,183 (70.5)
RAAS inhibitors	395 (34.9)	1,201 (38.8)
Statins	158 (14.0)	621 (20.1)

Appendix 6B. Baseline characteristics of subgroup population according to age

	Age <63 years (n = 1,989)	Age ≥63 years (n = 2,238)
Age, median (IQR), years	51 (44, 58)	72 (67, 77)
Female, No. (%)	504 (25.3)	628 (28.1)
Heart rate, median (IQR), beats/minute	77 (70, 85)	76 (69, 83)
SBP, median (IQR), mm Hg	116 (105, 128)	121 (110, 134)
DBP, median (IQR), mm Hg	69 (61, 78)	68 (60, 76)
Alcohol use, No. (%)	887 (44.6)	765 (34.2)
Smoking, No. (%)		
No	981 (49.3)	1,224 (54.7)
Current	448 (22.5)	316 (14.1)
Past	560 (28.2)	698 (31.2)
Peripheral edema, No. (%)	262 (13.2)	351 (15.7)
LVEF, median (IQR), %	32 (27, 37)	34 (28, 37)
Distribution, No. (%)		
30 to 40%	1,280 (64.4)	1,584 (70.8)
20 to 29%	621 (31.2)	598 (26.7)
≤19%	88 (4.4)	56 (2.5)
NYHA classification, No. (%)		
I	308 (15.5)	382 (17.1)
II	392 (19.7)	348 (15.5)
III	889 (44.7)	903 (40.3)
IV	400 (20.1)	605 (27.0)
eGFR, median (IQR), mL/min/1.73 m^2	91.79 (73.10, 104.11)	71.81 (55.80, 85.89)
BUN, median (IQR), mmol/L	6.60 (5.20, 8.50)	7.28 (5.60, 9.60)
Hemoglobin, median (IQR), g/L	136 (119, 150)	129 (114, 142)
NT-proBNP, median (IQR), pg/mL	12,044 (1,734, 35,000)	19,629 (2,214, 35,000)
HbA1c, median (IQR), %	5.17 (4.70, 6.10)	5.40 (4.75, 6.40)
Medical history, No. (%)		
Diabetes mellitus	358 (18.0)	584 (26.1)
Chronic obstructive pulmonary disease	142 (7.1)	581 (26.0)
Hypertension	523 (26.3)	1,018 (45.5)
Acute myocardial infarction	231 (11.6)	394 (17.6)
Ischemic heart disease	649 (32.6)	1,305 (58.3)
Rheumatic heart disease	242 (12.2)	149 (6.7)
Cardiomyopathy	790 (39.7)	434 (19.4)
Arrhythmia	770 (38.7)	907 (40.5)
Medical treatment, No. (%)		
Diuretics[†]	1,320 (66.4)	1,591 (71.1)

Beta-blockers	1,518 (76.3)	1,447 (64.7)
RAAS inhibitors	772 (38.8)	824 (36.8)
Statins	292 (14.7)	487 (21.8)

Appendix 6C. Baseline characteristics of subgroup population according to diabetes status

	Without diabetes (n = 3,285)	With diabetes (n = 942)
Age, median (IQR), years	63 (51, 72)	66 (58, 73)
Female, No. (%)	904 (27.5)	228 (24.2)
Heart rate, median (IQR), beats/minute	76 (70, 84)	76 (70, 84)
SBP, median (IQR), mm Hg	118 (107, 130)	122 (110, 135)
DBP, median (IQR), mm Hg	68 (60, 77)	70 (62, 78)
Alcohol use, No. (%)	1,285 (39.1)	367 (39.0)
Smoking, No. (%)		
No	1,729 (52.6)	476 (50.5)
Current	582 (17.7)	182 (19.3)
Past	974 (29.6)	284 (30.1)
Peripheral edema, No. (%)	450 (13.7)	163 (17.3)
LVEF, median (IQR), %	33 (28, 37)	33 (28, 37)
Distribution, No. (%)		
30 to 40%	2,220 (67.6)	644 (68.4)
20 to 29%	849 (28.9)	270 (28.7)
≤19%	116 (3.5)	28 (3.0)
NYHA classification, No. (%)		
I	512 (15.6)	178 (18.9)
II	586 (17.8)	154 (16.3)
III	1,413 (43.0)	379 (40.2)
IV	774 (23.6)	231 (24.5)
eGFR, median (IQR), mL/min/1.73 m^2	81.84 (64.91, 97.47)	73.08 (55.66, 88.90)
BUN, median (IQR), mmol/L	6.83 (5.30, 8.74)	7.40 (5.70, 10.00)
Hemoglobin, median (IQR), g/L	132 (116, 146)	132 (116, 145)
NT-proBNP, median (IQR), pg/mL	14,407 (1,948, 35,000)	23,680 (2,135, 35,000)
HbA1c, median (IQR), %	5.04 (4.65, 5.76)	6.94 (5.98, 8.10)
Medical history, No. (%)		
Chronic obstructive pulmonary disease	563 (17.1)	160 (17.0)
Hypertension	1,015 (30.9)	526 (55.8)
Acute myocardial infarction	423 (12.9)	202 (21.4)
Ischemic heart disease	1,311 (39.9)	643 (68.3)
Rheumatic heart disease	340 (10.4)	51 (5.4)
Cardiomyopathy	1,022 (31.1)	202 (21.4)
Arrhythmia	1,344 (40.9)	333 (35.4)
Medical treatment, No. (%)		
Diuretics[†]	2,225 (67.7)	686 (72.8)
Beta-blockers	2,292 (69.8)	673 (71.4)

RAAS inhibitors	1,184 (36.0)	412 (43.7)
Statins	528 (16.1)	251 (26.6)

Appendix 6D. Baseline characteristics of subgroup population according to eGFR

	eGFR <60 mL/min/1.73m^2 (n = 938)	eGFR ≥60 mL/min/1.73m^2 (n = 3,289)
Age, median (IQR), years	71 (63, 78)	61 (50, 70)
Female, No. (%)	286 (30.5)	846 (25.7)
Heart rate, median (IQR), beats/minute	76 (70, 82)	76 (70, 84)
SBP, median (IQR), mm Hg	120 (110, 134)	119 (107 130)
DBP, median (IQR), mm Hg	68 (60, 77)	68 (61, 77)
Alcohol use, No. (%)	314 (33.5)	1,338 (40.7)
Smoking, No. (%)		
No	540 (57.6)	1,665 (50.6)
Current	124 (13.2)	640 (19.5)
Past	274 (29.2)	984 (29.9)
Peripheral edema, No. (%)	162 (17.3)	451 (13.7)
LVEF, median (IQR), %	33 (27, 37)	33 (28, 37)
Distribution, No. (%)		
30 to 40%	620 (66.1)	2,244 (68.2)
20 to 29%	278 (29.6)	941 (28.6)
≤19%	40 (4.3)	104 (3.2)
NYHA classification, No. (%)		
I	171 (18.2)	519 (15.8)
II	114 (12.2)	626 (19.0)
III	367 (39.1)	1,425 (43.3)
IV	286 (30.5)	719 (21.9)
eGFR, median (IQR), mL/min/1.73 m^2	48.50 (41.33, 55.37)	87.26 (74.45, 99.18)
BUN, median (IQR), mmol/L	9.61 (7.40, 12.30)	6.40 (5.10, 8.10)
Hemoglobin, median (IQR), g/L	126 (110, 141)	134 (118, 147)
NT-proBNP, median (IQR), pg/mL	30,982 (2,837, 35,000)	11,781 (1,832, 35,000)
HbA1c, median (IQR), %	5.64 (4.85, 6.70)	5.20 (4.70, 6.11)
Medical history, No. (%)		
Diabetes mellitus	307 (32.7)	635 (19.3)
Chronic obstructive pulmonary disease	196 (20.9)	527 (16.0)
Hypertension	498 (53.1)	1,043 (31.7)
Acute myocardial infarction	182 (19.4)	443 (13.5)
Ischemic heart disease	547 (58.3)	1,407 (42.8)
Rheumatic heart disease	54 (5.8)	337 (10.2)
Cardiomyopathy	226 (24.1)	998 (30.3)
Arrhythmia	390 (41.6)	1,287 (39.1)
Medical treatment, No. (%)		
Diuretics[†]	756 (80.6)	2,155 (65.5)

Beta-blockers	617 (65.8)	2,348 (71.4)
RAAS inhibitors	415 (44.2)	1,180 (35.9)
Statins	220 (23.5)	559 (17.0)

† Diuretics included loop diuretics and spironolactone.

BUN: blood urea nitrogen; DBP: diastolic blood pressure; eGFR: estimated glomerular rate filtration; HbA1c: glycated hemoglobin; LVEF: left ventricular ejection fraction; NT-proBNP: N-terminal pro-B-type natriuretic peptide; NYHA: New York Heart Association; RAAS: renin-angiotensin-aldosterone system; SBP: systolic blood pressure.

Appendix 7. Results of subgroup analyses

	Outcomes (SGLT2 inhibitors plus standard therapy/standard therapy alone)					
	ICER ($/QALY)	Costs per person ($)	QALYs per person	Life years per person	Deaths	Total hospitalizations for heart failure
**Sex**						
**Females (n = 1,132)**	13,539.68	9,083.94/8,787.48	1.036/1.004	1.584/1.535	354/390	506/766
**Males (n = 3,095)**	9,365.53	9,035.05/ 8,731.42	1.051/1.018	1.576/1.526	981/1,081	1,372/2,072
**Age**						
**≥63 years (n = 2,238)**	10,132.82	9,129.28/8,826.99	1.049/1.017	1.594/1.545	686/759	990/1,497
**<63 years (n = 1,989)**	9,905.95	9,079.47/8,778.88	1.059/1.027	1.584/1.535	621/685	883/1,335
**Diabetes status**						
**With diabetes (n = 942)**	10,032.84	9,061.87/8,762.66	1.052/1.020	1.581/1.532	296/326	417/631
**Without diabetes (n = 3,285)**	9,470.90	9,042.89/8,742.96	1.045/1.013	1.577/1.528	1,039/1,144	1,459/2,206
**eGFR**						
**≥60 mL/min/1.73m² (n = 3,289)**	9,446.79	9,059.41/8,753.74	1.054/1.021	1.581/1.532	1,033/1,139	1,451/2,191
**<60 mL/min/1.73m² (n = 938)**	13,065.32	9,143.52/8,840.69	1.040/1.009	1.597/1.549	286/316	412/622

eGFR: estimated glomerular rate filtration; ICER: incremental cost-effectiveness ratio; QALYs: quality-adjusted life years; SGLT2: sodium-glucose cotransporter-2.

Appendix 8. Simulation results for subgroup analyses

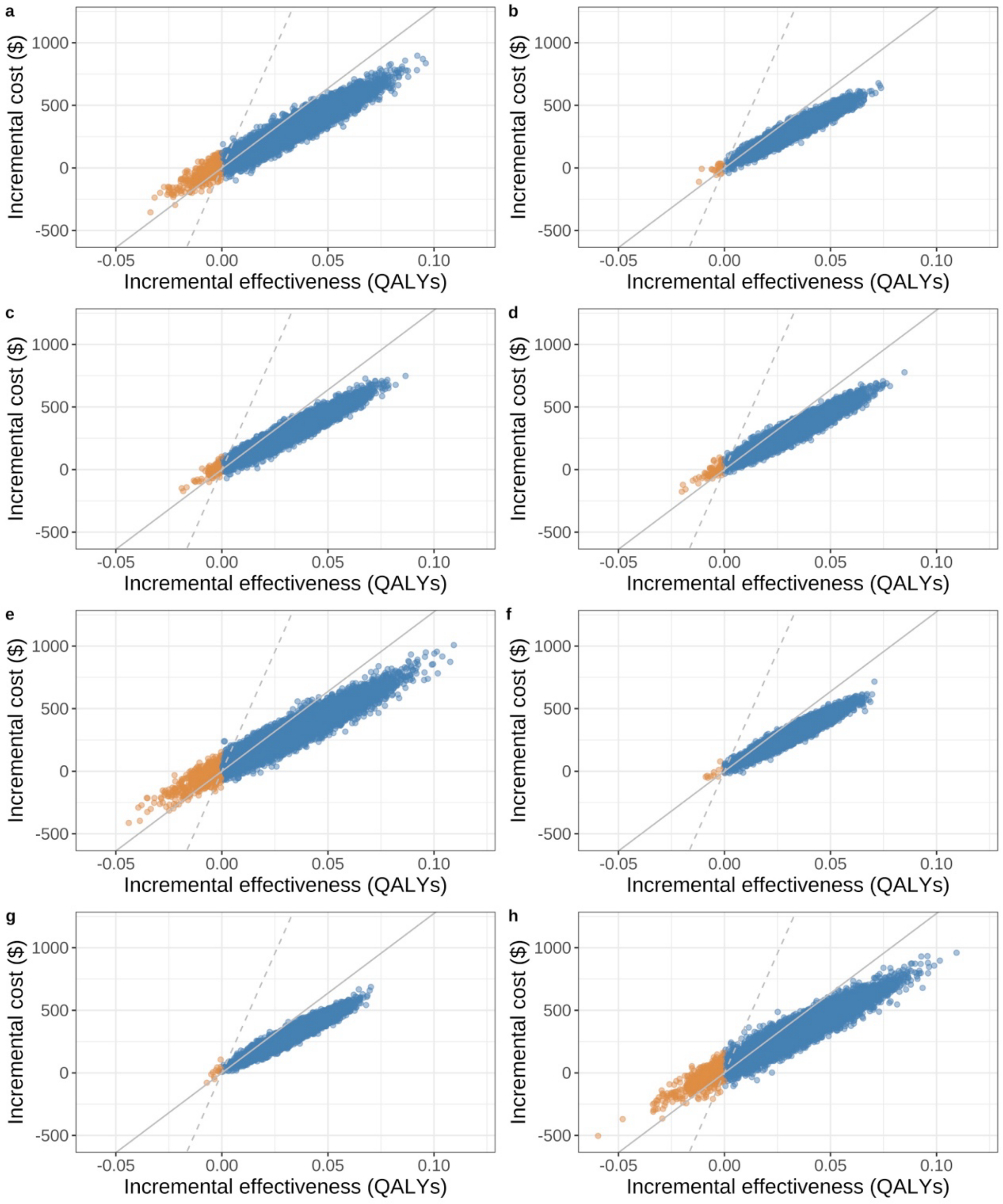

Appendix 8a-h shows the distribution of 10,000 results in the subgroup analyses in two years for subgroup population: (a) females; (b) males; (c) age <63 years; (d) age ≥63 years; (e) diabetes; (f) no diabetes; (g) eGFR ≥60 mL/min/1.73m²; (h) eGFR <60 mL/min/1.73m². The x-axis represents incremental QALYs per person between SGLT2 inhibitors plus standard therapy and standard therapy alone, and the y-axis represents incremental cost per person. The grey solid line represents the willingness-to-pay threshold of GDP per capita ($12,741/QALY), while the grey dashed line represents the willingness-to-pay threshold of three times GDP per capita ($38,223/QALY). The orange dots represent that SGLT2 inhibitors plus standard therapy were not more effective than standard therapy alone (incremental QALYs <0), the blue dots represent that SGLT2 inhibitors plus standard therapy were more effective than standard therapy alone (incremental QALYs >0).

QALYs: quality-adjusted life years; SGLT2: sodium-glucose cotransporter-2.

Appendix 9. Results of one-direction sensitivity analysis

	ICER at low value ($/QALY)	ICER at high value ($/QALY)	Range of input parameters
Transition probability for all-cause death	14,134.5	7,406.6	1/3 to 3 times the original value
Transition probability for first HHF	15,290.6	5,193	1/3 to 3 times the original value
Transition probability for second HHF	12,431.3	5,219.9	1/3 to 3 times the original value
Transition probability for third HHF	10,998.3	7,124.1	1/3 to 3 times the original value
Transition probability for fourth HHF	9,816.3	8,938.3	1/3 to 3 times the original value
Transition probability for genital infection	9,329.4	9,456.9	1/3 to 3 times the original value
Cost of standard HF therapy	4,759.3	24,775	1/3 to 3 times the original value
Cost of HHF per event	16,523.4	-12,112.7	1/3 to 3 times the original value
Cost of SGLT2 inhibitors	970.7	33,893.6	1/3 to 3 times the original value
Cost of genital infection per event	9,318.9	9,390.1	1/3 to 3 times the original value
Utility of NYHA class I HF	9,847.5	9,098.9	The original value ± 10%
Utility of NYHA class II HF	9,781.6	9,072.7	The original value ± 10%
Utility of NYHA class III HF	9,741.5	9,056.8	The original value ± 10%
Utility of NYHA class IV HF	9,377.8	9,049.8	The original value ± 10%
Disutility of first HHF	9,375.2	9,415.2	The original value ± 10%
Disutility of second HHF	9,320.8	9,359.5	The original value ± 10%
Disutility of third HHF	9,313.2	9,322.8	The original value ± 10%
Disutility of fourth HHF	9,302.1	9,280.3	The original value ± 10%
Disutility of genital infection	9,310.7	9,358.1	The original value ± 10%
RR for all-cause death in the first cycle	7,778.4	11,596.3	The original value ± 20%
RR for all-cause death in the second cycle	9,070.6	10,271.9	The original value ± 20%
RR for all-cause death in the third cycle	9,119.4	9,751.2	The original value ± 20%
RR for all-cause death in the fourth cycle	9,166.3	9,501.8	The original value ± 20%
RR for HHF in the first cycle	7,971.8	11,047.8	The original value ± 20%
RR for HHF in the second cycle	8,194.4	10,438	The original value ± 20%

RR for HHF in the third cycle	8,250.5	10,482.6	The original value ± 20%
RR for HHF in the fourth cycle	10,658	8,994.5	The original value ± 20%
RR for genital infection	9,337.1	9,363.8	The original value ± 20%
Discount rate for QALYs	8,947.2	9,974.8	0 to 8%
Discount rate for costs	9,753.9	8,754.4	0 to 8%

HF: heart failure; HHF: hospitalization for heart failure; ICER: incremental cost-effectiveness ratio; NYHA: New York Heart Association; QALY: quality-adjusted life year; RR: risk ratio; SGLT2: sodium-glucose cotransporter-2.

Appendix 10. Cost-effectiveness acceptability curve of the probabilistic sensitivity analysis

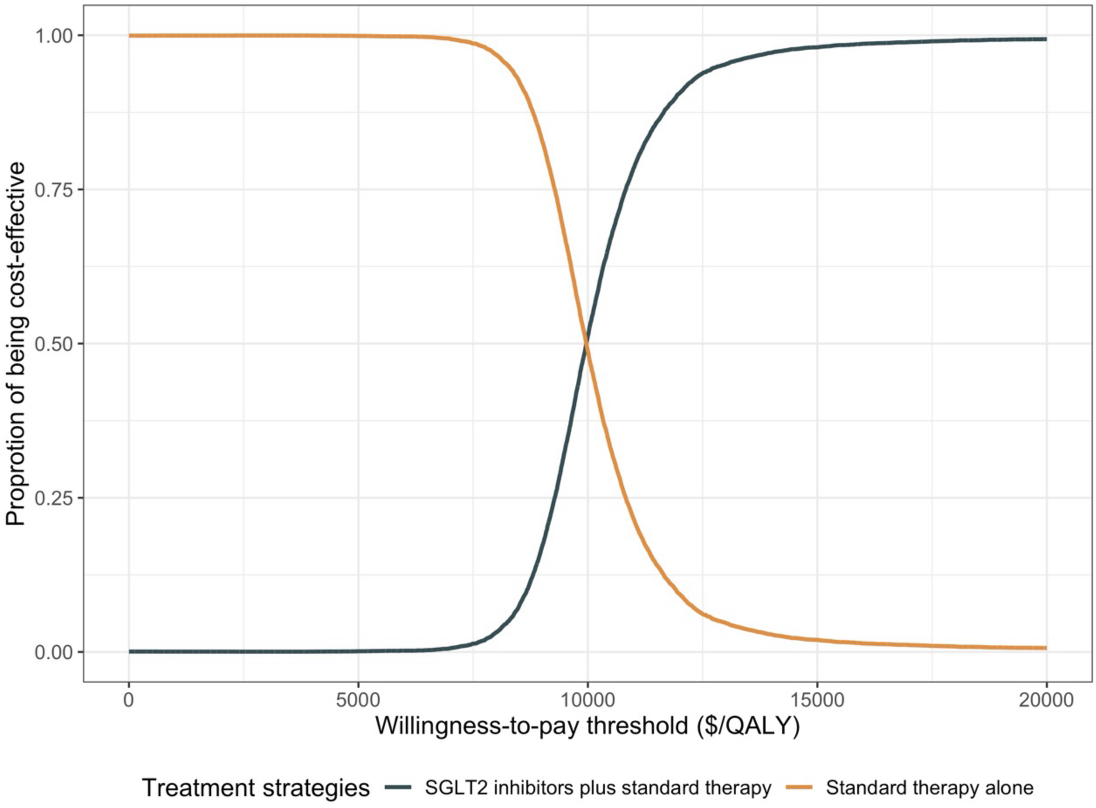

Appendix 10 shows the cost-effectiveness acceptability curve based on 10,000 results in the probabilistic sensitivity analysis.

QALYs: quality-adjusted life years; SGLT2: sodium-glucose cotransporter-2.

References

1 Levey AS, Stevens LA, Schmid CH, et al. A new equation to estimate glomerular filtration rate. *Ann Intern Med* 2009; **150:** 604–612.

2 Voors AA, Ouwerkerk W, Zannad F, et al. Development and validation of multivariable models to predict mortality and hospitalization in patients with heart failure. *Eur J Heart Fail* 2017; **19:** 627–634.

3 Huang X, Liu J, Hu S, et al. Systolic blood pressure at admission and long-term clinical outcomes in patients hospitalized for heart failure. *ESC Heart Fail* 2021; **8:** 4007–4017.

4 Hirji I, Andersson SW, Guo Z, Hammar N, Gomez-Caminero A. Incidence of genital infection among patients with type 2 diabetes in the UK General Practice Research Database. *J Diabetes Complications* 2012; **26:** 501–505.

5 Accessed January 30, 2024. https://www.scyxzbcg.cn/webPortal/home.html.

6 Huang J, Yin H, Zhang M, Ni Q, Xuan J. Understanding the economic burden of heart failure in China: impact on disease management and resource utilization. *J Med Econ* 2017; **20:** 549–553.

7 National Health Commission of the People's Republic of China. 2022 China Health Statistics Yearbook. Accessed January 30, 2024. http://www.nhc.gov.cn/mohwsbwstjxxzx/tjtjnj/202305/6ef68aac6bd14c1eb9375e01a0faa1fb.shtml.

8 National Bureau of Statistics of China. China Statistical Yearbook 2023. Accessed January 30, 2024. https://www.stats.gov.cn/sj/ndsj/2023/indexeh.htm.

9 Göhler A, Geisler BP, Manne JM, et al. Utility Estimates for Decision–Analytic Modeling in Chronic Heart Failure—Health States Based on New York Heart Association Classes and Number of Rehospitalizations. *Value Health* 2009; **12:**185–187.

10 Barry HC, Ebell MH, Hickner J. Evaluation of suspected urinary tract infection in ambulatory women: A cost-utility analysis of office-based strategies. *J Fam Pract* 1997; **44:** 49–60.
